# Mitochondrial Function in Gilles de la Tourette Syndrome Patients With and Without Intragenic *IMMP2L* Deletions

**DOI:** 10.3389/fneur.2020.00163

**Published:** 2020-03-24

**Authors:** Victoria A. Bjerregaard, Bitten Schönewolf-Greulich, Lene Juel Rasmussen, Claus Desler, Zeynep Tümer

**Affiliations:** ^1^Department of Clinical Genetics, Kennedy Center, Copenhagen University Hospital, Rigshospitalet, Denmark; ^2^Department of Cellular and Molecular Medicine, Center for Healthy Aging, University of Copenhagen, Copenhagen, Denmark; ^3^Department of Clinical Medicine, University of Copenhagen, Copenhagen, Denmark

**Keywords:** Gilles de la Tourette syndrome, GTS, *IMMP2L*, mitochondria, oxidative stress

## Abstract

**Background:** Gilles de la Tourette syndrome (GTS) is a neurodevelopmental condition characterized by motor and vocal tics. The underlying etiology remains largely unknown, and GTS is considered as a complex multifactorial disorder associated with effects of several genes in combination with environmental factors. The inner mitochondrial membrane peptidase, subunit 2 (*IMMP2L*) has been suggested as one of the susceptibility genes for GTS, and IMMP2L-deficient mouse and human cells show increased levels of mitochondrial oxidative stress and altered cell fate programming. Hence, a potential involvement of IMMP2L-induced mitochondrial dysfunction in GTS pathology is yet to be elucidated. To address this, we investigated mitochondrial function in a group of GTS patients with intragenic *IMMP2L* deletions and compared with GTS without *IMMP2L* deletions and healthy controls.

**Methods:** Mitochondrial function in fibroblasts from GTS patients and non-GTS parents (with and without *IMMP2L* deletions) compared to healthy controls were evaluated by measuring mitochondrial superoxide production, mitochondrial membrane potential, mitochondrial mass, and mitochondrial respiration. In addition, we evaluated apoptosis and senescence.

**Results:** None of the mitochondrial parameters assessed in this study were significantly distinctive when comparing GTS patients with and without *IMMP2L* deletions against healthy controls or parents with or without *IMMP2L* deletions, and we did not observe altered cell programming.

**Conclusion:** This study suggests that *IMMP2L* deletions do not lead to a substantial general mitochondrial dysfunction in GTS fibroblasts. Assessing a large cohort of controls and patients of similar age and gender would possibly reveal small differences in mitochondrial function. However, it is possible that *IMMP2L* variants affect mitochondrial function during specific instances of stress stimuli or in brain regions suggested to be affected in GTS.

## Introduction

Gilles de la Tourette syndrome (GTS) is a neurodevelopmental disorder, characterized by sudden, repetitive, non-rhythmic movements or sounds, referred to as tics. The GTS diagnosis is based on several motor and at least one vocal tic that have persisted for more than a year. There is significant comorbidity between GTS and other neurobiological/neuropsychiatric conditions, especially attention deficit hyperactivity disorder (ADHD) and obsessive–compulsive disorder (OCD). The disease etiology is complex and multifactorial, with an evident genetic component (1). One of the suggested GTS susceptibility genes is the inner mitochondrial membrane peptidase, subunit 2 (*IMMP2L*) (2–7), and structural variants involving this gene are also implicated in other neurobiological/neuropsychiatric conditions including autism and ADHD (8–11). *IMMP2L* transcript is expressed in several brain regions including the cerebellum (7). Cerebellum is implied to have a role not only in motor function, but also in cognitive and emotional processes, and its dysfunction is implicated both in movement disorders (e.g., ataxia and dystonia) and non-motor neuropsychiatric diseases (e.g., autism and ADHD) (12). As mitochondrial dysfunction is also linked to disorders affecting cerebellum (13), *IMMP2L* is a plausible susceptibility factor for neurobiological/neuropsychiatric disorders including GTS.

Studies in different organisms (yeast, mice, and human cells) show that substrates processed by IMMP2L include cytochrome c1, mitochondrial glycerol-3-phosphate dehydrogenase 2 (GPD-M), and apoptosis inducing factor (AIF) (14–16). Cytochrome c1 is involved in electron transfer in the mitochondrial electron transport chain, and GPD-M is a component of the mitochondrial glycerol phosphate shuttle, which functions in shuttling of electrons mitochondrial carriers in the oxidative phosphorylation pathway. Both peptides hence have important roles in mitochondrial respiratory chain and metabolism (17–19). AIF triggers apoptosis and promotes removal of damaged and irreparable cells when activated by IMMP2L under oxidative stress, while in the absence of IMMP2L, cells are driven toward a senescent state (16). Thus, IMMP2L has an important role both in both mitochondrial metabolism and cell-fate determination.

Brain tissue from Immp2l mutant mice show increased production of reactive oxygen species (ROS), hyperpolarization, and increased levels of ATP, but seem to have a normal volume of mitochondria and bioenergetic capacity (15, 20). Phenotypically, Immp2l mutant mice display a series of features including altered behavior, reduced social interaction, early onset ataxia, and age-dependent degeneration of cerebellar granule neurons (21–25). The majority of these phenotypes has been proposed to be a consequence of cytotoxic insults caused by an increased superoxide production.

Our group has previously identified intragenic *IMMP2L* deletions in a Danish cohort of GTS patients, and these deletions had an occurrence that was significantly high compared to Danish controls (frequency, 3.7 vs. 0.9%, respectively) (7). We hypothesized that impaired mitochondrial function through a defective cerebellar IMMP2L may contribute to GTS pathogenesis. To test this hypothesis, we conducted a series of mitochondrial studies in fibroblasts obtained from GTS patients with or without *IMMP2L* deletions compared to control fibroblasts obtained from parents without GTS and with or without deletions, and from asymptomatic controls. To our knowledge, this is the first study to evaluate mitochondrial function in fibroblasts from GTS patients.

## Methods

### Subjects and Study Design

In this study, we analyzed skin fibroblasts available from four of our previously published GTS patients (P1, P2, P5, P6) and three non-GTS parents (of P1, P2, P6) with *IMMP2L* deletions (7). According to their own descriptions, the three parents had some behavioral features at subclinical level (Table 1). Three anonymous GTS patients and four non-GTS controls (including mothers of P1 and P6) without deletions were also included in the study. The following considerations were made for the inclusion of family members: (1) inclusion of two asymptomatic mothers without deletions (of P1 and P6) in the control group would ensure similar mitochondrial DNA background when comparing fibroblasts with and without *IMMP2L* deletions; (2) inclusion of the parents with a deletion and without a GTS diagnosis (of P1, P2, P6) would minimize the effect of the background variation in the nuclear genome on mitochondrial function, as half of the genetic material in the nuclear genome is identical between child–parent.

**Table 1:** Subjects investigated in this study and information on fibroblast cultures.

**Fibroblast**	**Subject identification**	**Sex**	**Symptoms**	***IMMP2L* status (deleted exons)^**§**^**	***IMMP2L* status effected transcript^**§**^**	**Age^**#**^**	**Passage**
IMMP2L-1	P1	M	GTS, ADHD	Deletion (1a, 1b, 2, 3, 3a, 3b)	Long/short	23	3
IMMP2L-2	P1 father	M	Dyslexia^*^, Temper^*^	Deletion (1a, 1b, 2, 3, 3a, 3b)	Long/short	52	2
IMMP2L-3	P2	M	GTS, ADHD	Deletion (2, 3)	Long	20	3
IMMP2L-4	P2 mother	F	Tics^*^, OCD^*^	Deletion (2, 3)	Long	53	3
IMMP2L-5	P5	M	GTS	Deletion (3a, 3b)	Short	23	2
IMMP2L-6	P6	M	GTS, ADHD, OCD	Deletion (3a, 3b)	Short	19	2
IMMP2L-7	P6 father	M	Stubbornness^*^	Deletion (3a, 3b)	Short	62	3
TS-1		M	GTS	No deletion	–	22	3
TS-2		M	GTS	No deletion	–	24	3
TS-3		M	GTS	No deletion	–	21	3
Control-1	P1 mother	F	No symptoms	No deletion	–	50	2
Control-2	P6 mother	F	No symptoms	No deletion	–	56	2
Control-3	104027	F	No symptoms	No deletion	–	30	3
Control-4	104028	M	No symptoms	No deletion	–	45	3

§ *IMMP2L has two alternative transcripts differing at the 5′-end: The long transcript has exons 1a, 1b, 2, 3, 5–7 (ATG start codon in exon2) and the short transcript has exons 3a, 3b, 5–7 (ATG start codon in exon 3a) (7)*.

# *Age (years) when biopsy was taken*.

* *Subclinical symptoms according to individuals own description*.

*GTS, Gilles de la Tourette syndrome; ADHD, attention deficit hyperactivity disorder; OCB, obsessive–compulsive behavior; OCD, obsessive–compulsive disorder; M, male; F, female*.

All the experiments investigating mitochondrial dysfunction were repeated at least three times. Quantitative visual scoring was performed blinded to avoid bias.

### Cell Cultures

Fibroblasts were cultured in Dulbecco's modified Eagle's medium (DMEM) (Gibco) supplemented with 10% fetal bovine serum (FBS) (Gibco) and 1% penicillin/streptomycin, at 37°C in a humidified atmosphere with 5% CO_2_. Cells were routinely tested for mycoplasma.

### Superoxide Generation Assay

The level of mitochondrial ROS was quantified by measuring MitoSOX red (Molecular Probes, Invitrogen) using high-throughput microscopy (Nucleocounter 3000). Fibroblasts were incubated with 5 μM MitoSOX for 20 min at 37°C and washed once in phosphate-buffered saline (PBS) before harvest. Cells were harvested, resuspended in 10 μg/ml Hoechst 33342 (Tocris), incubated for 10 min at 37°C, and immediately analyzed. MitoSOX Red was excited at 530 nm, and data were collected at 675/75 nm. Hoechst was excited at 365 nm, and data were collected at 430/20 nm. Only live cells were included, and the mean fluorescence intensity (MFI) was obtained by subtracting the fluorescence of the control cells (not stained with MitoSOX) from the fluorescence of the MitoSOX stained cells. Antimycin A (150 μM) (Sigma-Aldrich) was used as a positive control. For each sample, a minimum of 5,000 cells were scored.

### Mitochondrial Membrane Potential

The mitochondrial membrane potential was determined by detecting tetramethylrhodamine, ethyl ester (TMRE) (Abcam) by high-throughput microscopy (Nucleocounter 3000). Fibroblasts were incubated with 100 nm TMRE for 15 min at 37°C, briefly washed in PBS, and harvested by standard procedures. Cells were resuspended in 10 μg/ml Hoechst 33342 (Tocris), incubated 10 min at 37°C, and immediately analyzed. TMRE was excited at 530 nm, and data were collected at 675/75 nm. Hoechst was excited at 365 nm, and data were collected at 430/20 nm. Only live cells were included in the MFI, and 20 μM carbonyl cyanide 4-(trifluoromethoxy) phenylhydrazone (FCCP) (Abcam) was used as a positive control. For each sample, a minimum of 5,000 cells were scored.

### Mitochondrial Mass

The mitochondrial mass of active mitochondria was quantified by measuring MitoTracker Green probe (Molecular Probes, Invitrogen) by high-throughput microscopy (Nucleocounter 3000). Cells were incubated with 100 nM MitoTracker for 20 min at 37°C and washed once in PBS before harvest. Cells were resuspended in 10 μg/ml Hoechst 33342 (Tocris), incubated 10 min at 37°C, and immediately analyzed. MitoTracker Green was excited at 475 nm, and data were collected in the 560/35 nm channel. Hoechst was excited at 365 nm, and data were collected in the 430/20 nm channel. Only live cells were included. For each sample, a minimum of 5,000 cells were scored.

### ATP Content and Mitochondrial Respiration

The ATP content was determined using the luciferase-based assay Vialight MDA Plus kit (Lonza) according to the manufacturer's instructions. Levels of luminescence was quantified in a Microbeta2 scintillation counter (Perkin Elmer).

The mitochondrial respiration was measured using an XF-96 Extracellular Flux Analyzer (Seahorse Bioscience, Agilent). Seeded cells were washed and resuspended in Seahorse assay media (Seahorse Bioscience, Agilent), supplemented with 1 mM pyruvate, 2 mM glutamine, and adjusted to pH 7.4. Oxygen consumption rates (OCRs) were measured to establish a baseline. Subsequently, wells were injected with either 1 μM oligomycin, to measure ATP turnover from the changes in OCR, or with 0.5 μM carbonyl cyanide *p*-(trifluoromethoxy) phenylhydrazone (FCCP) to determine reserve respiratory capacity from change in OCR. All cells were finally treated with 2 μM antimycin A as a control.

### Apoptosis and Senescence

The apoptotic state of the fibroblasts was evaluated using the Annexin V-CF488A conjugate (Biotium) following the instructions of the Nucleocounter NC-3000 Annexin V Assay (Application note no. 3017 Rev. 1.4). For each sample, a minimum of 5,000 cells were scored.

Senescence was analyzed using the SA-β-Gal-based senescence detection kit (Abcam) according to the manufacturer's instructions. The percentage of senescent cells were scored after five passages using the EVOS XL Core Cell Imaging System (Life Technologies) and the cell counter plugin ImageJ software (NIH). A minimum of 200 cells were counted per sample per experimental replica.

### Statistics

Results are presented as mean ± SD from at least three experimental replicas. A one-way analysis of variance (ANOVA) and Tukey tests were used to compare subjects individually or as specified groups (Table 1). A *p* < 0.05 was considered significant. All statistical analyses were carried out using Prism software (Graphpad).

## Results

The subjects investigated in this study were divided into three groups: (1) seven individuals with *IMMP2L* deletions (IMMP2L1–7), where four of them were clinically diagnosed with GTS (P1, P2, P5, and P6) and three of them were parents (of P1, P2, and P6) without a GTS diagnosis; (2) three GTS patients without *IMMP2L* deletions (TS 1–3); and (3) four controls without any GTS symptoms (control 1–4) including mothers of patients P1 and P6 (Table 1).

**Figure 1:**
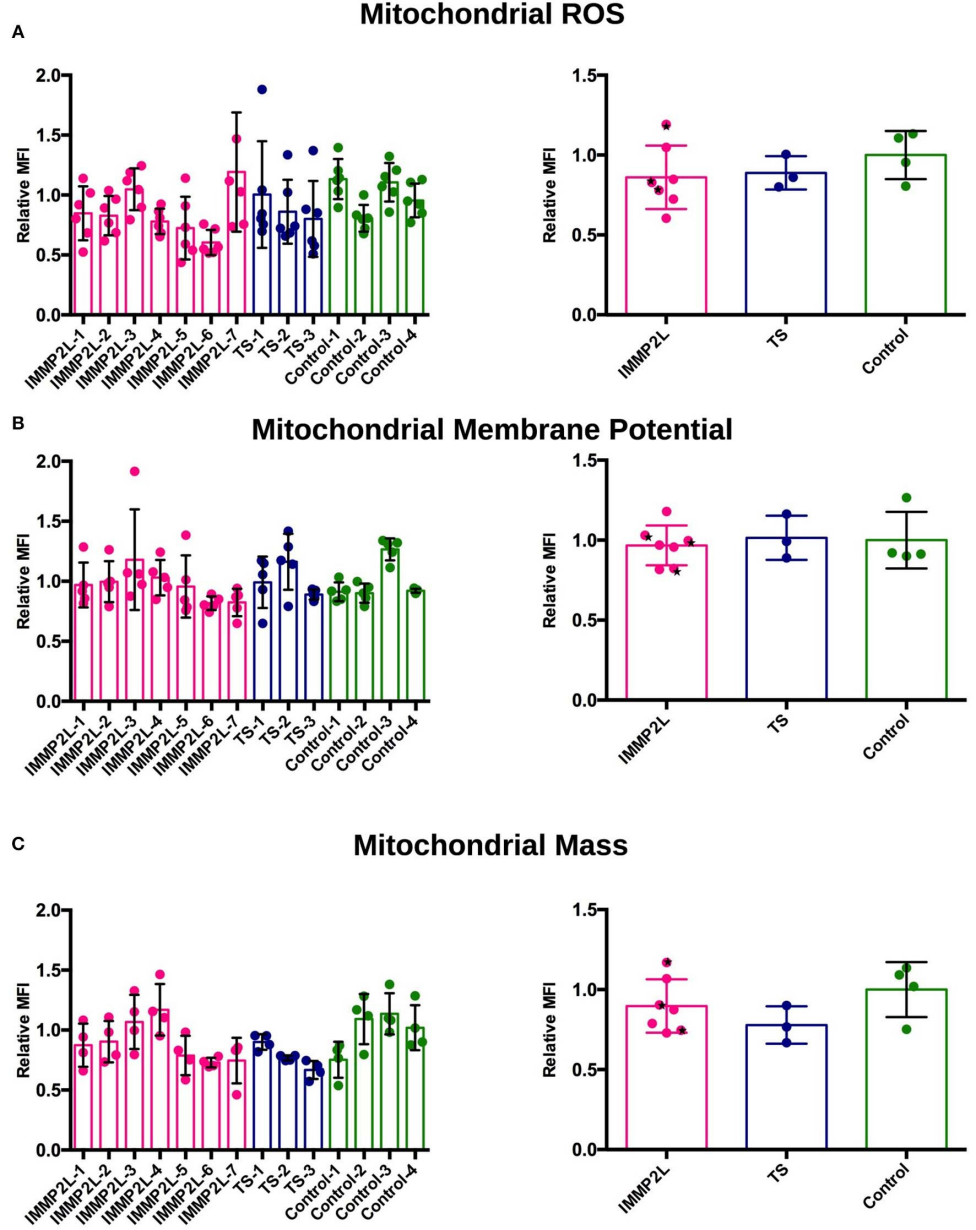
Fibroblasts of Gilles de la Tourette syndrome (GTS) patients with and without *IMMP2L* deletions display no signs of oxidative stress, altered membrane potential, or abnormal mitochondrial mass. **(A)** Mitochondrial reactive oxygen species (ROS) levels presented as relative MitoSOX MFI ± SD of each subject (left) and of each group (right). **(B)** Mitochondrial membrane potential presented as relative TMRE MFI ± SD of each subject (left) and of each group (right). **(C)** Mitochondrial mass presented as MitoTracker MFI ± SD of each subject (left) and of each group (right). All MFI values are normalized to the mean of the healthy control cells within each experimental replica. Each bar represents at least three independent experiments. Black stars mark non-GTS individuals with *IMMP2L* deletions.

To determine the level of mitochondrial ROS, we measured mitochondrial levels of superoxide, which represents total mitochondrial ROS, using mitoSOX in living and unstressed fibroblasts. In cells from all subjects, the level of ROS was below that of the stressed positive control (antimycin A), and we did not observe any significant differences between each subject or between the *IMMP2L* deletion, GTS-without deletion, or control group (Figure 1A). The results did not change when the non-GTS parents with or without *IMMP2L* deletions were omitted from the analysis (statistical analysis not included, raw data available).

Mitochondrial membrane potential was measured using the fluorophore TMRE that labels mitochondria proportional to the potential across the inner membrane. Depolarized or inactive mitochondria fail to sequester TMRE. The membrane potential of mitochondria is related to its ability to produce ATP by oxidative phosphorylation, but it is also an indicator of general apoptosis, as collapse of the mitochondrial membrane triggers an apoptotic cascade. No significant difference in mitochondrial membrane potential was demonstrated between fibroblasts of individual patients or between the three groups (Figure 1B).

Determination of mitochondrial mass provides a simplified overview of mitochondrial dynamics and is one of the signs of an altered activity of mitochondrial fission, fusion, biogenesis, or mitophagy. To label mitochondria in live cells, we used a MitoTracker probe, which passively diffuses across the plasma membrane and accumulates in active mitochondria. We did not observe significant difference in mitochondrial mass between individual subjects or between the three groups (Figure 1C).

**Figure 2:**
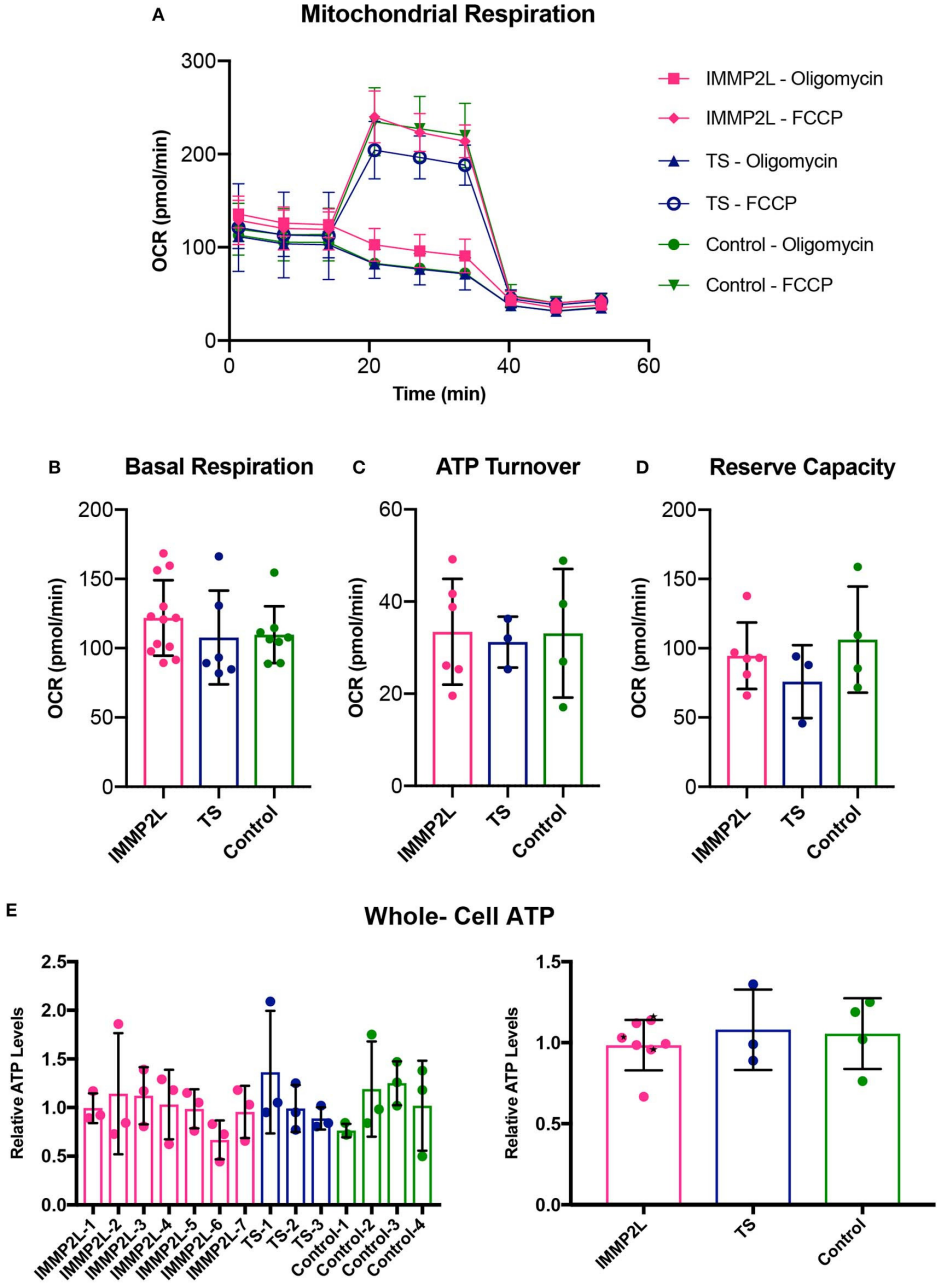
Properties of mitochondrial respiration were unaltered in fibroblasts of Gilles de la Tourette syndrome (GTS) patients with and without *IMMP2L* deletions. **(A)** Respiration overview of groups. **(B)** Basal respiration as determined as initial resting consumption of oxygen. **(C)** ATP turnover as measured as a decrease in oxygen consumption after addition of oligomycin. **(D)** Reserve respiratory capacity as measured as a percentage of basal respiration, after addition of FCCP. The similarity between the groups was also present in whole-cell ATP levels. **(E)** Mean percentage ± SD of whole-cell ATP presented for each subject (left) and the three groups (right). Black stars mark non-GTS individuals with *IMMP2L* deletions. All values are normalized to the mean of the healthy control cells within each experimental replica. Each bar represents at least three independent experiments.

To evaluate the bioenergetic capacity, we quantified basal respiration rate, ATP turnover, and reserve respiratory in live cells by standard protocols using the Seahorse XF analyzer (Figures 2A–D), and for a total overview of the energy metabolism, whole-cell count of ATP was determined (Figure 2E). None of the assays showed a significant difference between fibroblasts of individual patients or between different groups as described above.

**Figure 3:**
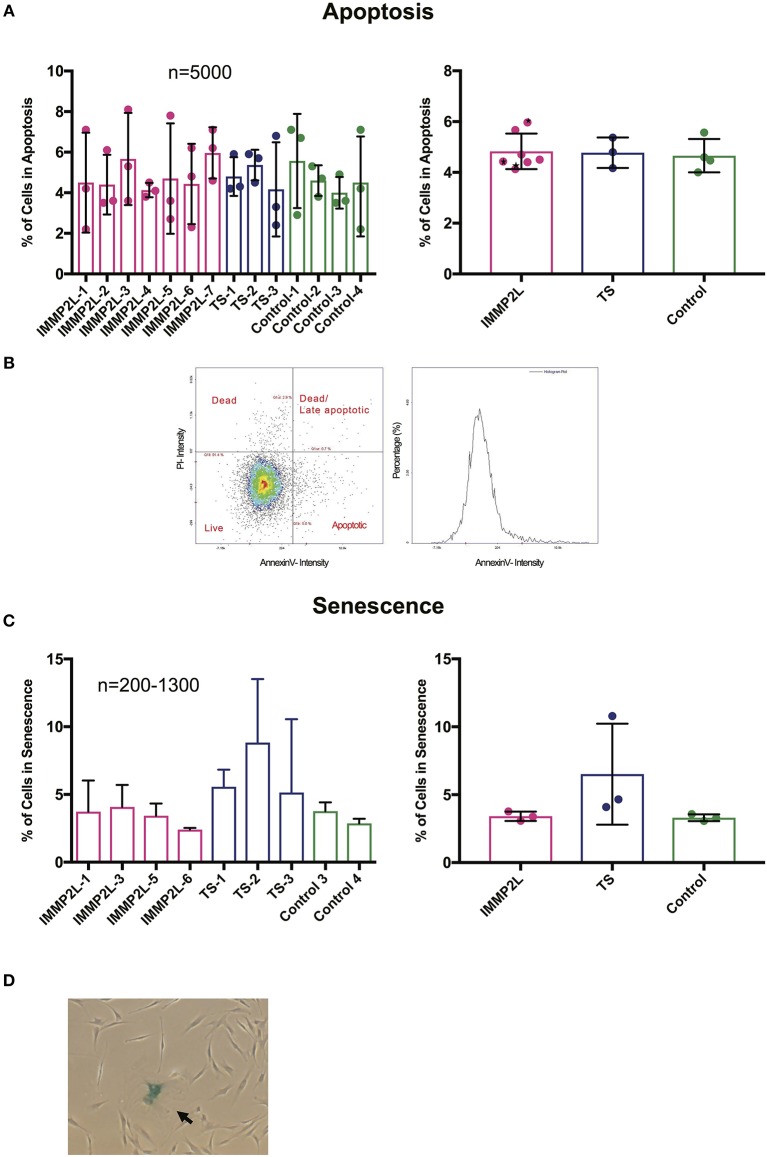
Gilles de la Tourette syndrome (GTS) patients with and without *IMMP2L* deletions show no signs of increased apoptosis or senescence. **(A)** Mean percentage ± SD of apoptotic cells presented for each subject (left) and the three groups (right). **(B)** Representatives of dot plot and histogram for Annexin V and propidium iodine (PI) staining. As indicated on the dot plot, the cell population was divided into apoptotic, late apoptotic/dead, and dead. The apoptotic population was included in the charts. Black stars mark non-GTS individuals with *IMMP2L* deletions. **(C)** Mean percentage ± SD of senescent cells presented for each subject (left) and the three groups (right). **(D)** Representative image of a fibroblast culture with a senescent cell positive for β-Gal (arrow). For all charts, each bar represents three independent experiments. *n* indicates the number of cells analyzed per replica.

To evaluate cell fate of the fibroblasts, we quantified the percentage of cells in apoptosis and senescence. Live apoptotic cells were stained with the apoptotic marker Annexin V and distinguished from dead cells by propidium iodine. The percentage of apoptotic cells were within a normal range for all fibroblasts, and we did not observe any differences between individuals or when comparing the two groups of GTS patients with controls (Figure 3A). To determine the percentage of cells in senescence, cells were fixed and stained with β-Gal in their exponential phase. Since the number of cells in senescence increases with age (26), individuals over 50 years of age were excluded from this assay (see Table 1). No significant difference was observed between individuals or between different groups as described above (Figure 3C).

## Discussion

In this study, we investigated whether mitochondrial dysfunction was a contributing factor in disease etiology of GTS patients with and without deletions affecting the mitochondrial peptidase IMMP2L. Having the highest mitochondrial energy demand of all organs, the brain in particular is sensitive to mitochondrial dysfunction, which indeed has been implicated in the etiology of a wide spectrum of neurobiological and neuropsychiatric disorders (27, 28). IMMP2L has a dual role in the mitochondria affecting both the mitochondrial metabolism and the cell fate. IMMP2L deficiency has been linked to mitochondrial dysfunction in the form of increased oxidative stress, a pathological feature common to several central nervous system disorders (29), and increased occurrence of cellular senescence, a pathological feature of neurodegeneration (30). In line with this, rare structural variants affecting *IMMP2L* were implicated as susceptibility factors in autism spectrum disorders (31) and ADHD (11).

*IMMP2L* has been suggested as a candidate susceptibility gene in GTS (3, 6), and we have shown that intragenic *IMMP2L* deletions were present at a higher frequency in GTS patients compared to control population. We hypothesized that the *IMMP2L* deletions could exert their effect through impaired mitochondrial function (7). To test this hypothesis, we conducted a series of experiments to assess whether fibroblasts from GTS patients with or without *IMMP2L* deletions would show phenotypes of oxidative stress or other signs of mitochondrial dysfunction compared to controls. As control individuals, we also included parents without GTS and with *IMMP2L* deletions, to ensure a more similar background for the nuclear genome to minimize any confounding effect of background variations, and asymptomatic mothers without deletions and to ensure a similar mitochondrial DNA background (Table 1). This would be an important issue in case we found a difference in mitochondrial function. However, we found no evidence for altered mitochondrial ROS, membrane potential, mass, respiration, or ATP content, suggesting that there is no substantial mitochondrial dysfunction in fibroblasts of GTS patients with or without *IMMP2L* deletions. However, these findings do not exclude that processing of known IMMP2L substrates, such as cytochrome c1, GPD-M, and AIF is affected, and further studies are necessary to clarify this.

IMMP2L has a key role in cell-fate programming during conditions of increased ROS by either promoting apoptosis by AIF activation or being shut down upon the onset of senescence (16). In GTS fibroblasts, we did not observe induction of either apoptosis or senescence, but this could be due to the normal ROS levels that we have measured. It is therefore plausible that in high ROS levels, a defective IMMP2L may not be able to process AIF to its proapoptotic active form. Further studies may elucidate how fibroblasts with *IMMP2L* deletions would respond to exogenous stimulation of ROS.

Cerebellum has been implicated in GTS pathophysiology (32, 33), and our group has previously shown that *IMMP2L* transcripts were highly expressed in the granular and Purkinje cell layer of the cerebellum (7). Abnormal or dysfunctional cerebellum and Purkinje cells have also been implicated in other neurodevelopmental and movement disorders, such as autism spectrum disorders, ataxia, and dystonia, and notably, mitochondrial dysfunction is linked to these disorders (34–36). It is thus possible that *IMMP2L* deletions have a more pronounced effect in high energy-dependent neurons, such as the Purkinje cells, which are known to be vulnerable to mitochondrial dysfunction (36). Notably, Purkinje cells are a class of GABAergic (γ-aminobutyric acid) neurons, and altered GABA function has been suggested to contribute to GTS pathology (37). Further studies using induced pluripotent stem cell (iPSC)-derived brain cells, including GABAergic neurons, are necessary to understand the involvement of mitochondrial dysfunction in brain tissues.

In summary, we could not show a substantial mitochondrial dysfunction in GTS patients with or without *IMMP2L* deletions in fibroblast. However, involvement of IMMP2L in GTS pathogenesis cannot be completely excluded, and further studies investigating larger number of patients and using iPSC-derived neuronal cells are necessary.

## Data Availability Statement

All datasets generated for this study are included in the article/supplementary material.

## Ethics Statement

This study was approved by the Danish Regional Committee on Health Research Ethics (H-1-2014-109). All included participants gave their specific consent to participate and for the publication of the data.

## Author Contributions

VB designed and carried out the experiments presented in Figures 1, 3. CD designed and conducted the experiments presented in Figure 2, with assistance from VB. BS-G collected the skin biopsies from patients. VB and CD performed the data analysis and interpretation. ZT conceptualized the project and guided the experimental design. LJ contributed with equipment and reagents. The manuscript was written by VB and ZT with the contribution of CD.

### Conflict of Interest

The authors declare that the research was conducted in the absence of any commercial or financial relationships that could be construed as a potential conflict of interest.
